# Exploring the Pharmacological Mechanism of Quercetin-Resveratrol Combination for Polycystic Ovary Syndrome: A Systematic Pharmacological Strategy-Based Research

**DOI:** 10.1038/s41598-019-54408-3

**Published:** 2019-12-05

**Authors:** Kailin Yang, Liuting Zeng, Tingting Bao, Zhiyong Long, Bing Jin

**Affiliations:** 1 Department of Cardiac Surgery, Beijing Anzhen Hospital, Capital Medical University, Beijing, China; 2 Capital Medical University, Beijing, China; 3 Xiyuan Hospital, China Academy of Chinese Medical Sciences, Beijing, China; 4 School of Clinical Medicine (Xiyuan Hospital), Beijing University of Chinese Medicine, Beijing, China; 5 Shantou University Medical College, Shantou University, Shantou, Guangdong, China

**Keywords:** Reproductive disorders, Medical research

## Abstract

Resveratrol and quercetin have effects on polycystic ovary syndrome (PCOS). Hence, resveratrol combined with quercetin may have better effects on it. However, because of the limitations in animal and human experiments, the pharmacological and molecular mechanism of quercetin-resveratrol combination (QRC) remains to be clarified. In this research, a systematic pharmacological approach comprising multiple compound target collection, multiple potential target prediction, and network analysis was used for comparing the characteristic of resveratrol, quercetin and QRC, and exploring the mechanism of QRC. After that, four networks were constructed and analyzed: (1) compound-compound target network; (2) compound-potential target network; (3) QRC-PCOS PPI network; (4) QRC-PCOS-other human proteins (protein-protein interaction) PPI network. Through GO and pathway enrichment analysis, it can be found that three compounds focus on different biological processes and pathways; and it seems that QRC combines the characteristics of resveratrol and quercetin. The in-depth study of QRC further showed  more PCOS-related biological processes and pathways. Hence, this research not only offers clues to the researcher who is interested in comparing the differences among resveratrol, quercetin and QRC, but also provides hints for the researcher who wants to explore QRC’s various synergies and its pharmacological and molecular mechanism.

## Introduction

Polycystic ovary syndrome (PCOS) is one of the most common female endocrine diseases characterized by hyperandrogenism, menstrual disorders and infertility. The prevalence of PCOS is estimated at 6–18%^1,2^. PCOS is associated with a variety of factors, such as systemic inflammation, endothelial dysfunction, endocrine and metabolic disorders and dyslipidemia^3,4^. Currently, lots of evidence has shown that PCOS is usually associated with hyperlipidemia, decreased glucose tolerance, cardiovascular disease, type 2 diabetes, cancer and hypertension^5,6^. In clinical, the main pharmacology options of PCOS are anti-androgen, oral contraceptives and so on. However, the treatment of PCOS remains a major challenge in obstetrics and gynecology. For example, metformin, as a first-line treatment for PCOS, can reduce peripheral blood glucose and insulin levels; however, long-term use may result in side effects such as gastrointestinal discomfort.

Although the etiology of PCOS is still unclear, plenty of evidence has suggested that hyperandrogenism and inflammation are the key factors^7–10^. Evidence also suggests that resveratrol and quercetin, the nature polyphenolic compounds, may be the potential novel drugs for hyperandrogenism. Latest clinical trials have found that resveratrol can significantly reduce ovarian and adrenal androgens, and improve insulin sensitivity^11^. Animal experiments also show that in PCOS rat models, resveratrol can improve plasma anti-Mullerian hormone levels, IGF-1 levels and oxidative stress parameters; and it also reduces rat theca-interstitial cell growth *in vitro* and inhibits insulin-induced rat theca-interstitial cell growth^12,13^. Another clinical trial about quercetin shows that quercetin effectively improves adiponectin - mediated insulin resistance^14^. And the animal experiments also suggest that in PCOS rat models, quercetin can inhibit Toll-like receptor/NF-κB signaling pathway and improve the ovarian inflammatory microenvironment (Interleukin [IL] 1β, IL-6 and tumor necrosis factor [TNF] -α). Moreover, quercetin can also inhibit phosphoinositide 3-kinase (PI3K) inhibition and decrease CYP17A1 gene expression^15^.

According to these references, it appears that resveratrol combined with quercetin may integrate the efficacy of both and become a new pharmacology option. However, because the targets from animal experiments are not as accurate as that from human experiments, while human experiments have some limitations, such as ethical restrictions and unable to exactly probe the molecular changes in the ovaries, the molecular mechanism of resveratrol and quercetin are still unclarified; it is, thus, hard to reveal the pharmacological and molecular mechanism of quercetin-resveratrol combination (QRC). Fortunately, with the rapid development of bioinformatics, systematic pharmacology was born, which combines computational and experimental methods^16^. Compared with previous methods, systematic pharmacology provides a synthesized “systems-level” methodology to determining the mechanisms of drugs in human patients and in animal models^16^. In other words, this methodology can explore the pharmacological and molecular mechanisms by “multi-drug - multi-target - multi-pathway - multi-directional” way^17^. Because of such characteristic, systematic pharmacology is widely used in drug discovery, target prediction and mechanism research^18^. Therefore, this research will utilize this systematic pharmacological methodology to explore the pharmacological and molecular mechanism of QRC, and compare the effects and characteristic of quercetin, resveratrol and QRC.

## Materials and Methods

### Materials preparation

#### Chemical structure acquisition

The chemical structures of “quercetin” and “resveratrol” were collected from SciFinder (http://scifinder.cas.org) and drawn with ChemBioDraw. Then, they were saved as “mol2” file.

#### Compound targets collection and potential targets prediction

The targets of quercetin and resveratrol were collected from Traditional Chinese Medicine Systematic pharmacology Database (TCMSP^TM^, http://ibts.hkbu.edu.hk/LSP/tcmsp.php). The potential targets of quercetin and resveratrol were predicted by PharmMapper (http://lilab-ecust.cn/pharmmapper/).

TCMSP^TM^ is a unique system pharmacology platform designed for natural compounds of herbs^19^. PharmMapper is a web server for potential drug target identification using pharmacophore mapping approach^20^.

#### Protein name correction

**Figure 1:**
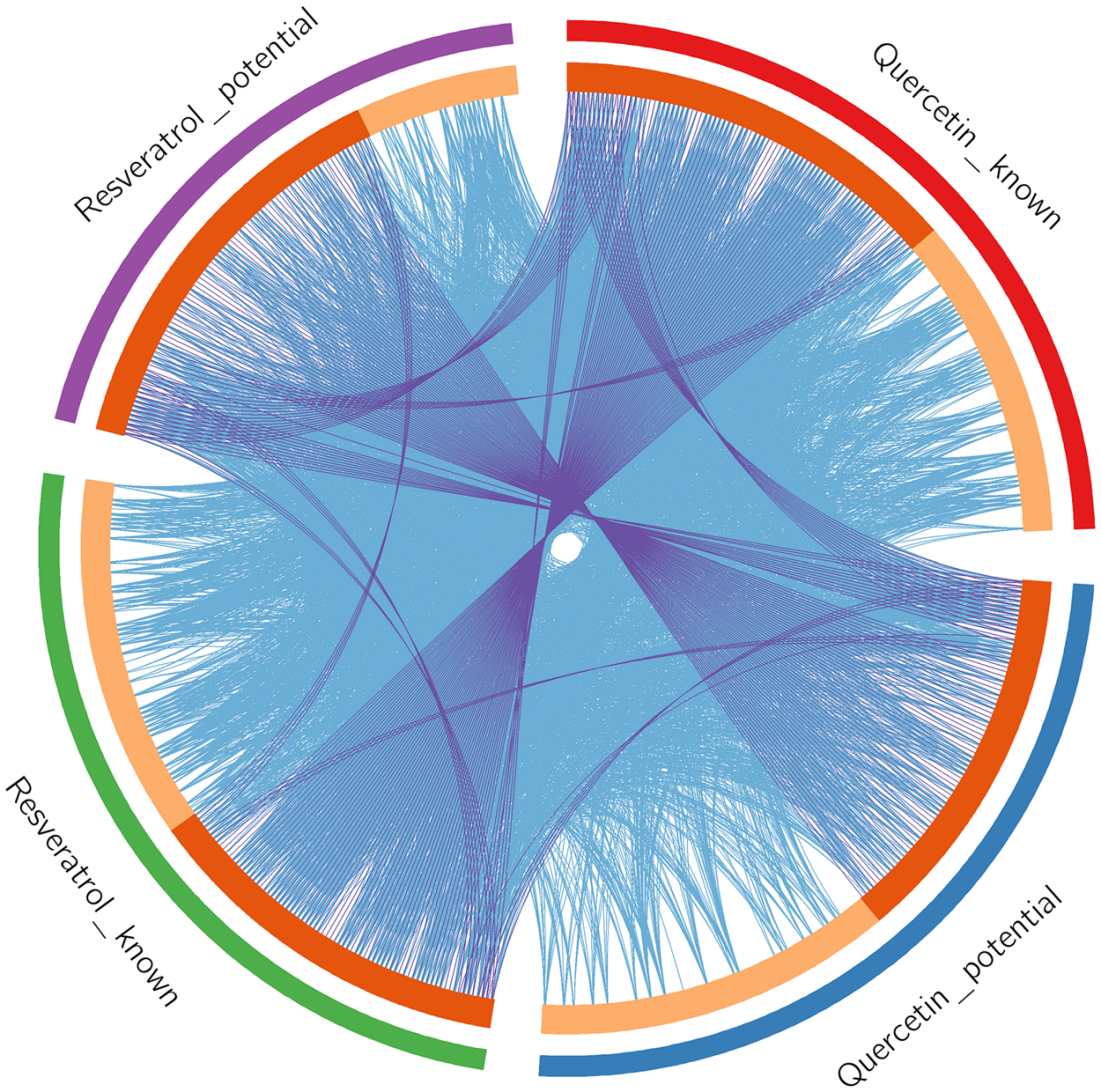
The circos plot of known and potential targets distribution.

Since the protein name of the collected target is not standard, they were corrected using UniProtKB (http://www.uniprot.org/) so as to obtain their official symbols. The details of these targets are described in Table S1 and Table S2. Metascape was utilized to exhibit distribution of known and potential targets (Fig. 1). In the outer circle, red and green represent known targets for quercetin and resveratrol, respectively, while blue and purple represent potential targets for quercetin and resveratrol, respectively. In the inner circle, the greater the number of purple links and the longer the dark orange arc, the more overlap between the input target lists. The blue link indicates the amount of functional overlap between the input target lists.

#### PCOS targets

Genecards (http://www.genecards.org) and OMIM database (http://omim.org/)^21^ were utilized to collect the PCOS genes. The keywords is “Polycystic Ovarian Syndrome”, “Polycystic Ovary Syndrome”, “Sclerocystic Ovarian Degeneration”, “Stein Leventhal Syndrome”, “Sclerocystic Ovary Syndrome” and get 420 genes totally. The details are described in Table S3.

#### Protein-protein interaction data

String database (http://string-db.org/, ver. 11) is used to collect the data of protein-protein interaction (PPI)^22^. String is a database of known and forecasted protein-protein interactions. When collecting PPI data, the species were limited to “Homo sapiens” with a confidence score >0.4.

### Gene ontology and pathway enrichment analysis

The DAVID ver. 6.8 (https://david-d.ncifcrf.gov) is applied for Gene Ontology (GO) enrichment analysis and pathway enrichment analysis^23^.

### Network and heatmap construction

#### Network construction method

The Cytoscape software was utilized to construct the networks (http://cytoscape.org/, ver. 3.4.0)^24^. Four networks were constructed: (1) compound-compound target network; (2) compound-potential target network; (3) QRC-PCOS PPI network; (4) QRC-PCOS-other human proteins PPI network.

#### Heatmap construction method

A heatmap is a graphical representation of data where the individual values contained in a matrix are represented as colors. We apply Heatmap Illustrato (http://hemi.biocuckoo.org/, ver. 1.0) to construct heatmap. Input the P-value of GO enrichment and pathway enrichment and get the heatmap of three compounds.

#### Cluster

The definition the acquisition methods of clusters were described in our previous work^25^. The clusters of each network were obtained by analyzing the corresponding networks by MCODE, a plug-in of Cytoscape^25,26^.

## Results and Discussion

### Compound-compound target network analysis

#### Network

**Figure 2:**
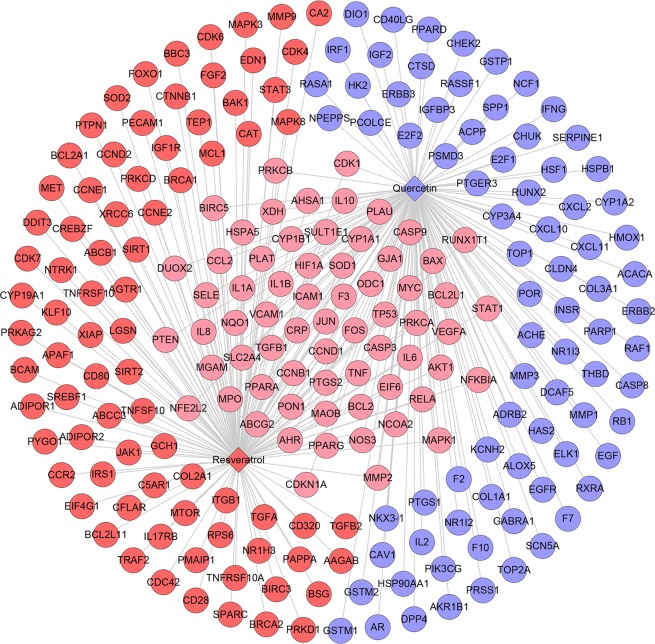
Compound-compound target network (Blue and red circles stand for compound targets of resveratrol and quercetin, resp. Pink circles stand for common compound targets of both resveratrol and quercetin. Red and blue diamonds stand for resveratrol and quercetin, resp).

This network consists of 227 nodes (225 compound target nodes and 2 compound nodes) and 291 edges (Fig. 2). In this network, twenty-six nodes in the center are regulated by both resveratrol and quercetin, while the peripheral nodes are hit by one of them. These nodes regulated by both two may be the central nodes or key nodes in treating diseases such as PCOS.

This network suggests that the quercetin and resveratrol may act on these compound targets and thus play a pharmacological role in other diseases such as PCOS. Their potential effect can be carried out by this network.

### Heatmap

Through GO enrichment, we get the biological processes about PCOS. P-value is Modified Fisher Exact P-Value, EASE score^23^. The smaller, the more enriched. Each biological process is marked by a GO identifier (GO ID) such as “GO: 0043066”. We can search QuickGO (http://www.ebi.ac.uk/QuickGO) with GO IDs to get the information of these biological processes. By comparing P-value, we can learn the main biological processes each compound focuses. The P-values of each biological process are shown in Table 1. The details of each biological process are shown in Table S4.

**Table 1:** P-value of biological processes.

Biological Processes	P-value		
	Resveratrol	Quercetin	QRC
Negative regulation of apoptotic process	1.62E-21	7.65E-13	9.28E-22
Positive regulation of cell proliferation	8.70E-11	9.41E-12	5.95E-17
Response to estradiol	5.97E-09	1.62E-11	9.88E-13
Inflammatory response	6.93E-10	1.15E-12	1.12E-12
Positive regulation of NF-kappab transcription factor activity	1.70E-09	2.02E-07	2.68E-10
Response to progesterone	9.64E-07	1.90E-05	3.68E-08
Positive regulation of I-kappab kinase/NF-kappab signaling	1.35E-06	4.59E-04	2.38E-07
Response to insulin	5.81E-09	0.002551	2.40E-07
Androgen receptor signaling pathway	0.005288	0.005088	1.02E-06
Response to estrogen	0.002401	1.88E-05	2.25E-06
Cellular response to interleukin-1	3.37E-05	2.37E-06	4.42E-06
Removal of superoxide radicals	2.50E-06	1.26E-04	1.38E-05
Ovarian follicle development	1.52E-06	1.40E-06	1.80E-05
Negative regulation of oxidative stress-induced intrinsic apoptotic signaling pathway	2.14E-04	0.006128	2.73E-05
Transforming growth factor beta receptor signaling pathway	1.45E-04	0.001134	3.05E-05
Insulin receptor signaling pathway	5.70E-04	—	7.87E-05
Steroid hormone mediated signaling pathway	0.086916	1.22E-04	1.06E-04
Response to oxidative stress	0.002653	3.56E-04	6.63E-04
Response to interleukin-1	0.032833	0.032008	0.070858

Tip: “—” means this formula does not have this pathway.

**Figure 3:**
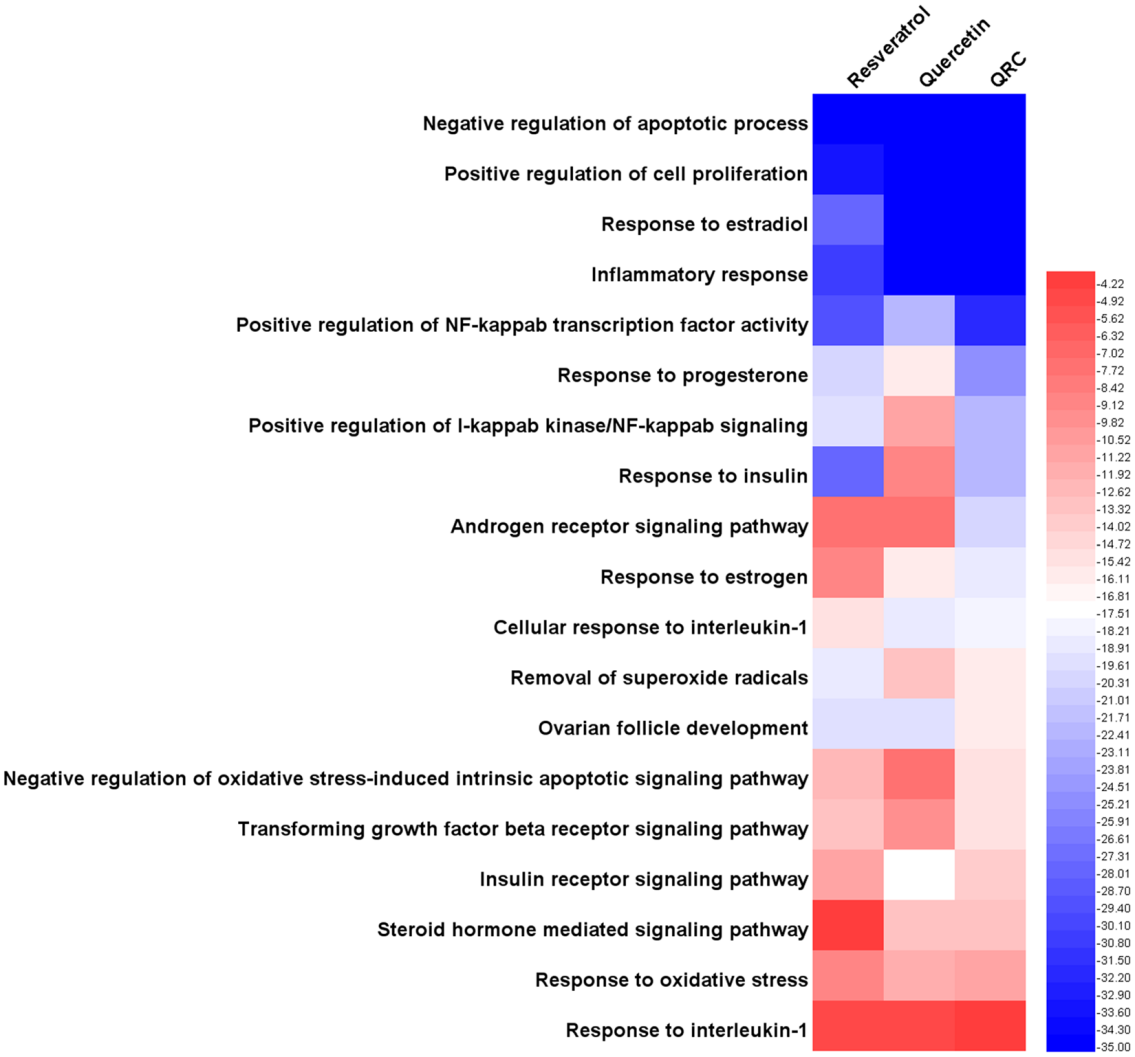
Heatmap of biological processes (The bluer, the more enriched. The blank means that this formula does not have this biological process).

In Fig. 3, we can find that the main biological processes of resveratrol, quercetin, QRC relating to treating PCOS are a little different. Though all of resveratrol, quercetin, QRC major in (GO: 0043066) negative regulation of apoptotic process, they have different degrees of effect on other biological processes (reflected as the P-value of enrichment). For example, resveratrol is better at GO: 0006954, GO: 0051092, GO: 0032868, GO: 0032355, GO: 0032570, GO: 0043123, GO: 0001541, GO: 0019430 and so on in sequence, while quercetin focuses on GO: 0006954, GO: 0008284, GO: 0032355, GO: 0051092, GO: 0001541, GO: 0071347, GO: 0043627, GO: 0032570 and so on in sequence. Also, QRC commit to GO: 0008284, GO: 0032355, GO: 0006954, GO: 0051092, GO: 0032570, GO: 0043123, GO: 0032868, GO: 0030521, GO: 0043627, GO: 0071347, GO: 0019430 and so on in sequence (Fig. 3).

Through pathway enrichment, we can know the main pathway each compound focuses by comparing P-value. Each pathway is marked by an identifier such as “hsa00980”. We can search Kyoto Encyclopedia of Genes and Genomes (KEGG, http://www.genome.jp/kegg/) with these identifiers to get the information of these pathways. The P-values of each pathway are shown in Table 2. The details of each pathway are shown in Table S5.

**Table 2:** P-value of pathways.

Pathways	P-value		
	Resveratrol	Quercetin	QRC
Apoptosis	2.02E-17	9.56E-10	6.99E-18
Insulin resistance	7.76E-13	2.79E-05	1.43E-11
NF-kappa B signaling pathway	5.23E-10	4.86E-07	1.92E-09
Insulin signaling pathway	4.82E-05	0.003949	2.93E-07
Estrogen signaling pathway	4.90E-04	8.22E-05	2.06E-05
GnRH signaling pathway	2.75E-04	0.001415	4.70E-05
Progesterone-mediated oocyte maturation	0.005537	0.005333	7.50E-04
Ovarian steroidogenesis	0.012846	0.012512	0.002442
Steroid hormone biosynthesis	0.093629	0.022073	0.023722
TGF-beta signaling pathway	0.020024	0.069899	0.031518
Oocyte meiosis	0.052679	—	0.088872

Tip: “—” means this formula does not have this pathway.

**Figure 4:**
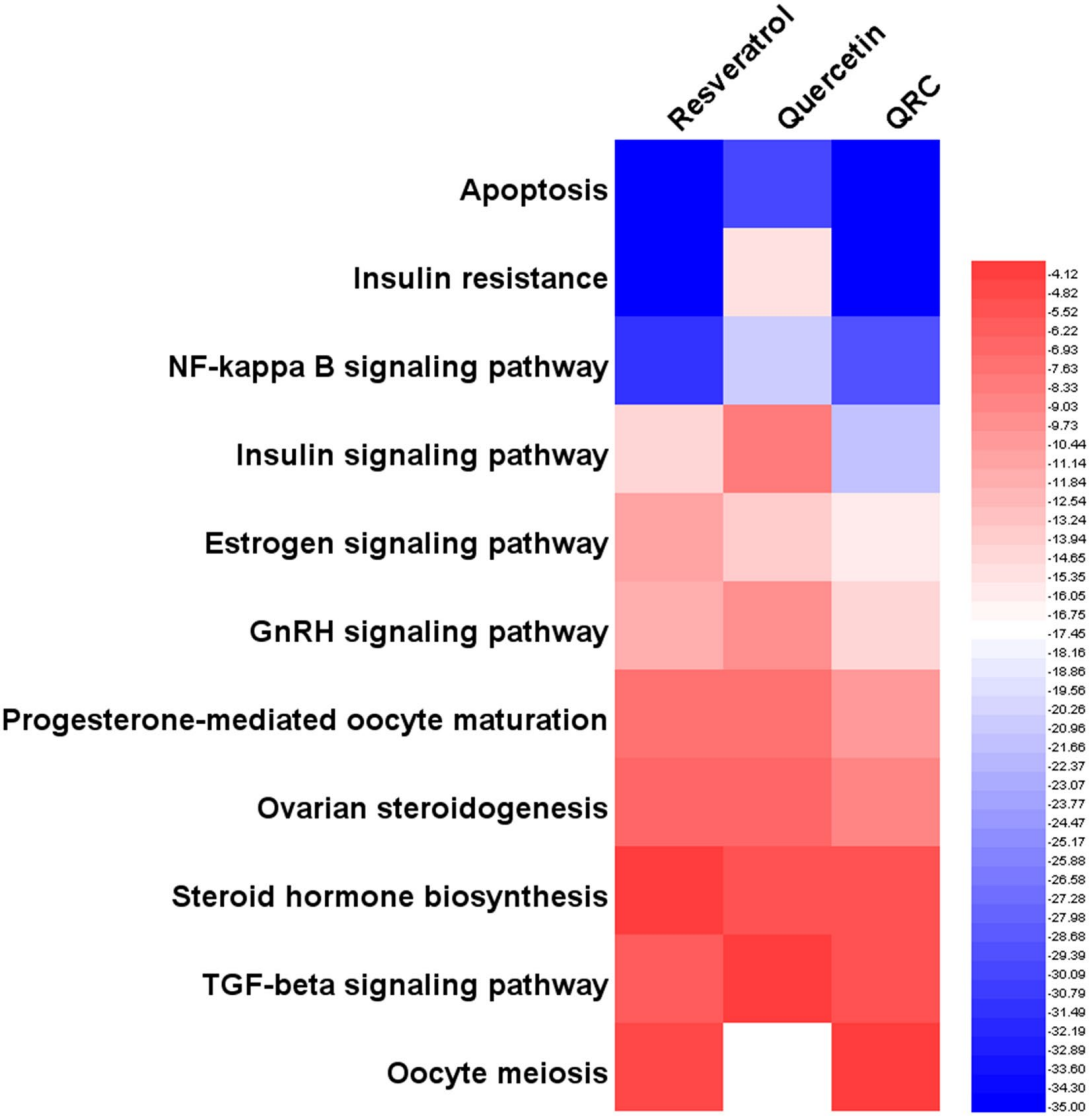
Heatmap of pathways (The bluer, the more enriched. The blank means that this formula does not have this pathway).

Through Fig. 4, we can know that although all of resveratrol, quercetin, QRC can regulate (hsa00140) steroid hormone biosynthesis and (hsa04210) apoptosis in sequence, the main pathways of them that associated with treating PCOS are not completely the same. For instance, resveratrol major in (hsa04931) insulin resistance, (hsa04064) NF-kappa B signaling pathway, (hsa04910) insulin signaling pathway, (hsa04912) GnRH signaling pathway, (hsa04915) estrogen signaling pathway, *et al*. in sequence; quercetin focuses on (hsa04064) NF-kappa B signaling pathway, (hsa04931) insulin resistance, (hsa04915) estrogen signaling pathway, (hsa04912) GnRH signaling pathway, *et al*. in sequence; QRC commit to (hsa04931) insulin resistance, (hsa04064) NF-kappa B signaling pathway, (hsa04910) insulin signaling pathway, (hsa04915) estrogen signaling pathway, *et al*. in sequence (Fig. 4).

Through heatmap, PCOS-associated biological processes and pathways that resveratrol, quercetin, QRC act on are different. It seems that we can make horizontal comparisons. For instance, in biological processes, for (GO: 0008284) positive regulation of cell proliferation, the P-value of QRC is the biggest, quercetin’s is smaller and resveratrol’s is the smallest, which likely shows that QRC is the most effective one in acting on cell proliferation; in pathways, (hsa04912) GnRH signaling pathway, the P-value of quercetin is the biggest, QRC’S is the smallest, which tends to demonstrate that QRC is the most effective one in regulating GnRH signaling pathway. However, due to the P-value calculation method, the P-value between different samples is quite different; thus, we cannot make a horizontal comparison. But, we can observe which biological processes and pathways the compounds tend to regulates (their sequence), which indicates that we can make a vertical comparison. In addition, based on current evidence, because of the limitations of databases, we can only illustrate the mechanisms and provide a theoretical basis for similar study. Meanwhile, we expect a new parametric or statistical algorithm to make the biological processes and pathways between the samples able to carry out a horizontal comparison. We also expect further proteomics and genomics research to complement and further validate our results.

### Compound-potential target network analysis

#### Network

**Figure 5:**
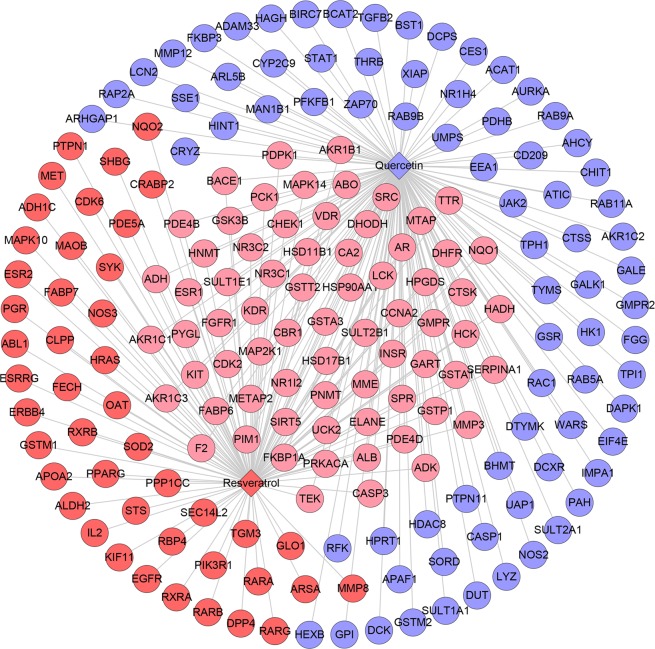
Compound-potential target network (Blue and red circles stand for compound targets of resveratrol and quercetin, resp. Pink circles stand for common compound targets of both resveratrol and quercetin. Red and blue diamonds stand for resveratrol and quercetin, resp).

Due to the limitations of compound targets, we predict the potential targets of each compound. All compounds, potential targets and the interactions are presented in Fig. 5. This network contains 193 nodes (191 compound target nodes and 2 compound nodes) and 261 edges (Fig. 5). It is smaller than the compound-compound target network. This is because that the compound targets come from experiments and some of them are indirectly regulated by resveratrol or quercetin, while the potential compounds can be directly regulated by them.

#### Heatmap

Get the PCOS-related biological processes from GO enrichment. By comparing P-value, we can know the main biological processes they focus. The P-values of each biological process are shown in Table 3. The details of each biological process are shown in Table S6.

**Table 3:** P-value of biological processes.

Biological Processes	P-value		
	Resveratrol	Quercetin	QRC
Steroid hormone mediated signaling pathway	1.00E-13	1.18E-04	4.25E-14
Negative regulation of apoptotic process	1.21E-09	1.30E-04	7.67E-09
Oxidation-reduction process	3.92E-05	1.29E-07	4.55E-08
Steroid metabolic process	0.002936	6.66E-08	4.36E-07
Positive regulation of cell proliferation	7.89E-08	0.002042	4.81E-06
Response to estrogen	4.61E-06	0.002226	7.66E-06
Cellular response to insulin stimulus	1.24E-05	4.88E-04	2.37E-05
Negative regulation of I-kappab kinase/NF-kappab signaling	0.029168	0.004653	9.95E-04
Cyclooxygenase pathway	0.001916	0.007424	0.003486
Progesterone metabolic process	0.058465	0.002459	0.004218
Insulin receptor signaling pathway	0.095555	0.003056	0.005234
Regulation of inflammatory response	—	0.001984	0.005319
Estrogen metabolic process	—	0.003715	0.006351
Removal of superoxide radicals	0.002786	—	0.007565
Testosterone biosynthetic process	0.026416	0.033402	0.04381
Regulation of cytokine production involved in inflammatory response	—	0.033402	0.04381
Response to estradiol	0.00321	0.042919	0.069895

Tip: “—” means this formula does not have this pathway.

**Figure 6:**
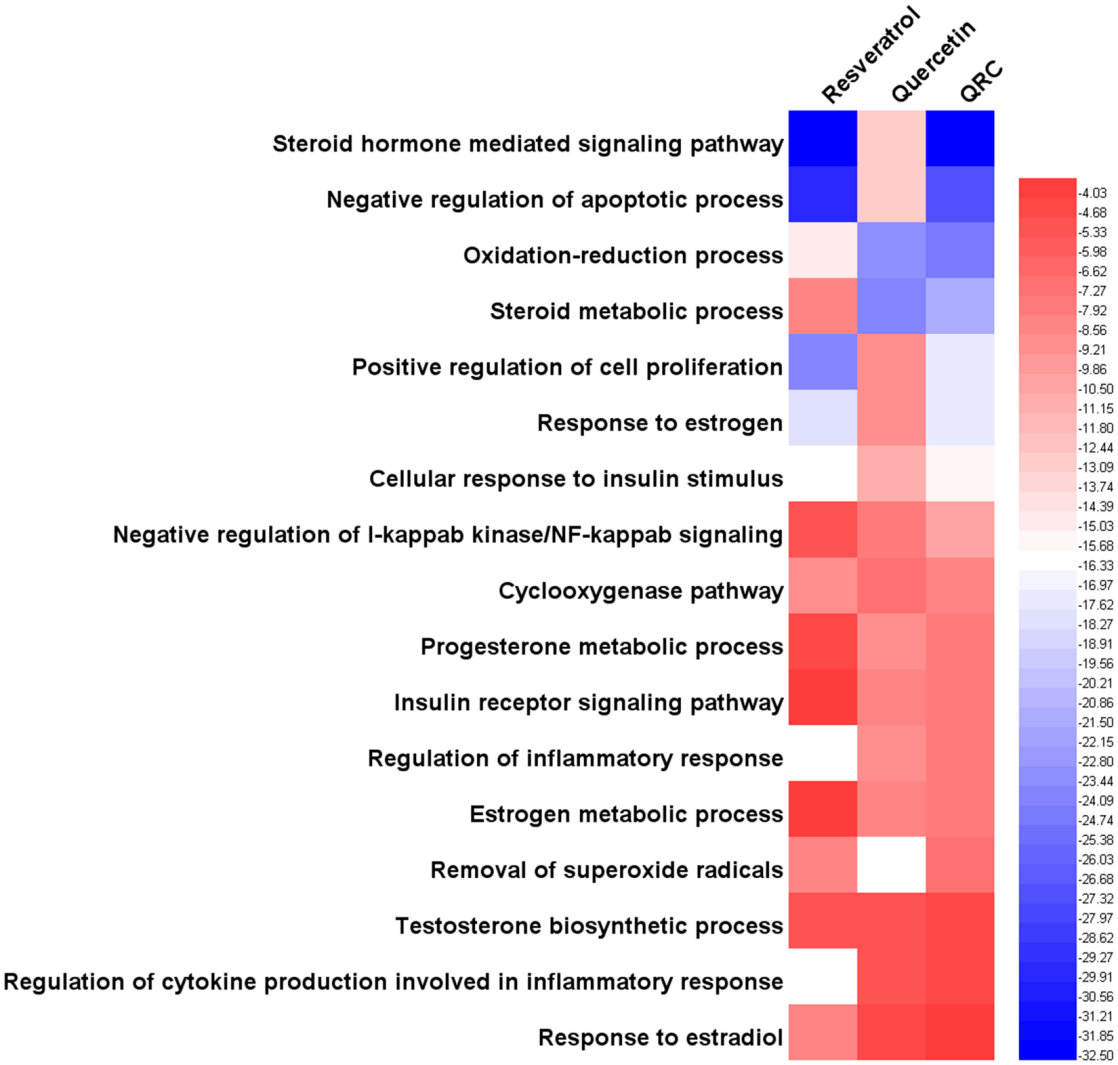
Heatmap of biological processes (The bluer, the more enriched. The blank means that this formula does not have this biological process).

Figure 6 shows that the main biological processes of resveratrol, quercetin, QRC that relate to treating PCOS are not completely the same. For instance, resveratrol focuses on GO: 0043401, GO: 0043066, GO: 0008284, GO: 0043627, GO: 0032869, GO: 0055114, GO: 0019371, *et al*. in sequence. Quercetin major in GO: 0008202, GO: 0055114, GO: 0043401, GO: 0043066, GO: 0032869, GO: 0050727, GO: 0008284, *et al*. in sequence. QRC commit to GO: 0043401, GO: 0043066, GO: 0055114, GO: 0008202, GO: 0008284, GO: 0043627, GO: 0032869, GO: 0043124, *et al*. in sequence (Fig. 6).

Through pathway enrichment, we can know the main pathway each compound focuses. The P-values of each pathway are shown in Table 4. The details of each pathway are shown in Table S7.

**Table 4:** P-value of pathways.

Pathways	P-value		
	Resveratrol	Quercetin	QRC
Metabolism of xenobiotics by cytochrome P450	7.34E-06	9.46E-07	1.14E-07
Insulin signaling pathway	2.80E-06	0.001049	1.86E-05
Insulin resistance	1.66E-05	0.050995	2.04E-04
Steroid hormone biosynthesis	1.39E-04	6.89E-04	3.76E-04
Estrogen signaling pathway	8.16E-06	0.037278	4.88E-04
Progesterone-mediated oocyte maturation	2.45E-05	0.022937	9.32E-04
GnRH signaling pathway	0.001584	0.089872	0.01962
Oocyte meiosis	0.017507	—	0.042446

Tip: “—” means this formula does not have this pathway.

**Figure 7:**
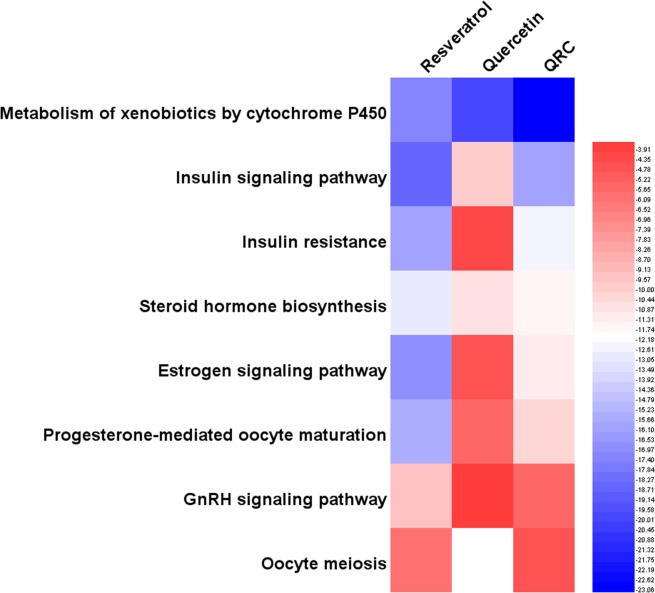
Heatmap of pathways (The bluer, the more enriched. The blank means that this formula does not have this pathway).

In Fig. 7, we can find that the main pathways of resveratrol, quercetin and QRC that associated with treating PCOS are not completely the same, too. For example, resveratrol major in (hsa04910) insulin signaling pathway, (hsa00980) metabolism of xenobiotics by cytochrome P450, (hsa04915) estrogen signaling pathway, (hsa04931) insulin resistance, (hsa04914) progesterone-mediated oocyte maturation, (hsa00140) steroid hormone biosynthesis, *et al*. in sequence; quercetin focuses on (hsa00980) metabolism of xenobiotics by cytochrome P450, (hsa00140) steroid hormone biosynthesis, (hsa04910) insulin signaling pathway, (hsa04914) progesterone-mediated oocyte maturation, *et al*. in sequence; QRC commit to (hsa00980) metabolism of xenobiotics by cytochrome P450, (hsa04910) insulin signaling pathway, (hsa04931) insulin resistance, (hsa00140) steroid hormone biosynthesis, (hsa04915) estrogen signaling pathway, (hsa04914) progesterone-mediated oocyte maturation, *et al*. in sequence (Fig. 7).

From previous figures, we can know that the main biological processes and pathways of resveratrol, quercetin and QRC are different; whether resveratrol, quercetin or QRC, they all have their own characteristics in regulating biological processes and pathways—they regulate which in different sequence. For example, for biological processes in potential targets’ network, resveratrol focuses on GO: 0043401, GO: 0043066, GO: 0008284 and so on in sequence, while quercetin major in GO: 0008202, GO: 0055114, GO: 0043401 and so on in sequence; also, QRC commit to GO: 0043401, GO: 0043066, GO: 0055114 and so on in sequence; for pathways in potential targets’ network, resveratrol major in (hsa04910) insulin signaling pathway, (hsa00980) metabolism of xenobiotics by cytochrome P450, (hsa04915) estrogen signaling pathway, *et al*. in sequence; quercetin focuses on (hsa00980) metabolism of xenobiotics by cytochrome P450, (hsa00140) steroid hormone biosynthesis, (hsa04910) insulin signaling pathway, *et al*. in sequence; QRC commit to (hsa00980) metabolism of xenobiotics by cytochrome P450, (hsa04910) insulin signaling pathway, (hsa04931) insulin resistance, *et al*. in sequence. Meanwhile, through these figures, it seems that QRC combines the features of resveratrol and quercetin. For instance, QRC contains more targets than resveratrol or quercetin—QRC includes both resveratrol and quercetin’s targets; and the biological processes and pathways QRC regulates is the combination of those of resveratrol and quercetin. Furthermore, there may be much more biological processes and pathways that can be found by further analysis and study.

According to current evidence, the possible mechanism of PCOS is thalamus - pituitary - ovarian axis abnormality (such as hyperandrogenism), hyperinsulinemia and insulin resistance, adrenal endocrine dysfunction, and systemic multiple systems abnormality.

The pathological manifestations of polycystic ovary are increased sinus follicles and ovarian matrix, as well as follicular hyperplasia (overexpression of follicles) and thickening of the ovarian cortex^3,26^. More studies have shown that the number of primary follicles in anovulatory polycystic ovary is significantly increased, while the proportion of primordial follicles is correspondingly reduced^27^. In addition, the polycystic ovary arteries are reduced compared to normal ovarian follicles^28^. These factors ultimately lead to disordered growth of primordial follicles and impaired dominant follicles^27–30^. This research shows that QRC can regulate several follicular developmental signaling pathways and biological processes, such as (hsa04914) progesterone-mediated oocyte maturation, (hsa04114) oocyte meiosis, (GO:0008284) positive regulation of cell proliferation.

Serum levels of androgen in patients with PCOS are high^31,32^. Increased androgen production may be due to increased frequency of gonadotropin-releasing hormone (GnRH) release, which triggers increased secretion of luteinizing hormone (LH), while LH stimulates ovarian follicular cells to produce testosterone. In addition, due to the increased activity of various steroidogenic enzymes in PCOS^33^, PCOS follicular cells secrete androgen and respond to LH and insulin^34^. This research shows that QRC can influence sex hormone-related signaling pathways and biological processes, such as (hsa04910) Insulin signaling pathway, (hsa04931) Insulin resistance, (hsa00140) Steroid hormone biosynthesis, (hsa04915) Estrogen signaling pathway, (hsa04914) Progesterone-mediated oocyte maturation, (hsa04912) GnRH signaling pathway, (GO:0008202) Steroid metabolic process, (GO:0008210) Estrogen metabolic process, (GO:0032355) Response to estradiol, (GO:0032570) Response to progesterone.

Hyperinsulinemia and insulin resistance also play a role in PCOS. Such fasting hyperinsulinemia is a combined effect of increased basal insulin secretion and decreased hepatic insulin clearance^35,36^. Current research indicates that insulin and insulin signaling pathways play an important role in controlling ovulation; some insulin effects on the ovary may be mediated by insulin-like growth factor (IGF)-I and insulin-IGF-I receptors^37^. Insulin increases glucose uptake by increasing the transport of the insulin-responsive glucose transporter GLUT4 from the intracellular vesicles to the cell surface^38,39^. This leads to obstacles in the glucose metabolism pathway and amplification of the mitogen-activated protein pathway, and the expression levels of mitogen-activated protein kinases and proliferating cell nuclear antigen molecules are elevated, eventually resulting in hyperproliferation of ovarian granulosa cells^40,41^. When the insulin receptor substrate signal transduction in ovarian tissue is in equilibrium, the insulin effect can be normalized; while this mechanism of patients with PCOS is unbalanced, leading to metabolic disorders and reproductive function disorders, which have a dual impact on hyperinsulinemia^42^. This research shows that QRC may affect insulin-related signaling pathways and biological processes, such as (hsa04910) insulin signaling pathway, (hsa04931) insulin resistance, (GO:0032869) cellular response to insulin stimulus.

In addition, hyperandrogenism, hyperinsulinemia and other factors may lead to obesity metabolic characteristics of PCOS^43,44^. Obesity in PCOS is associated with chronic low-grade inflammation (mediators including TNF-α, high-sensitivity C-reactive protein and IL)^45,46^, in which TNF-α mediates insulin resistance^47^. This research shows that QRC can also affect inflammation, such as (hsa00980) metabolism of xenobiotics by cytochrome P450, (GO:0019371) cyclooxygenase pathway, (GO:0043124) negative regulation of I-kappab kinase/NF-kappab signaling.

In summary, the main biological processes and pathways of resveratrol, quercetin or QRC that relate to treating PCOS are different (Figs. 3, 4, 6, 7 and Tables 1–4). The new biological processes and pathways that have not appeared in the compound targets’ enrichment analysis may be the potential ones needing more experiment and deep study to confirm, and may be the main direction of future research. In the treatment of PCOS, these biological processes and pathways may be the key ones; and the genes and proteins (compound targets and potential targets) in key biological processes and pathways may be the central genes and central proteins. Through this comparison, it appears that QRC combines the characteristics of resveratrol and quercetin and may have the potential to be a new pharmacology option for the treatment of PCOS. We decide to analyze its network and study its pharmacological and molecular mechanisms in depth.

### The In-depth analysis of QRC

**Figure 8:**
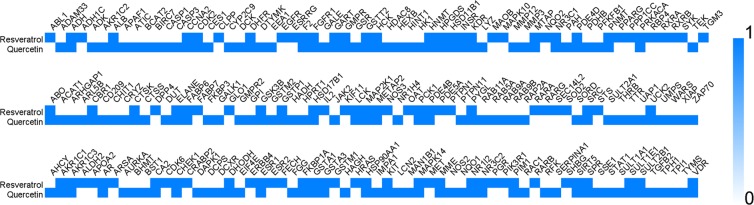
The targets resveratrol, quercetin or both of them regulate.

Because of that the pharmacological and molecular mechanism of QRC has not been completely clarified, we need an in-depth analysis. And through Fig. 8 we can understand the targets that resveratrol, quercetin or both of them regulate. The P-value 0 stands for that the compound does not regulate this target, while the P-value 1 stands for a converse situation.

#### QRC-PCOS PPI network

**Figure 9:**
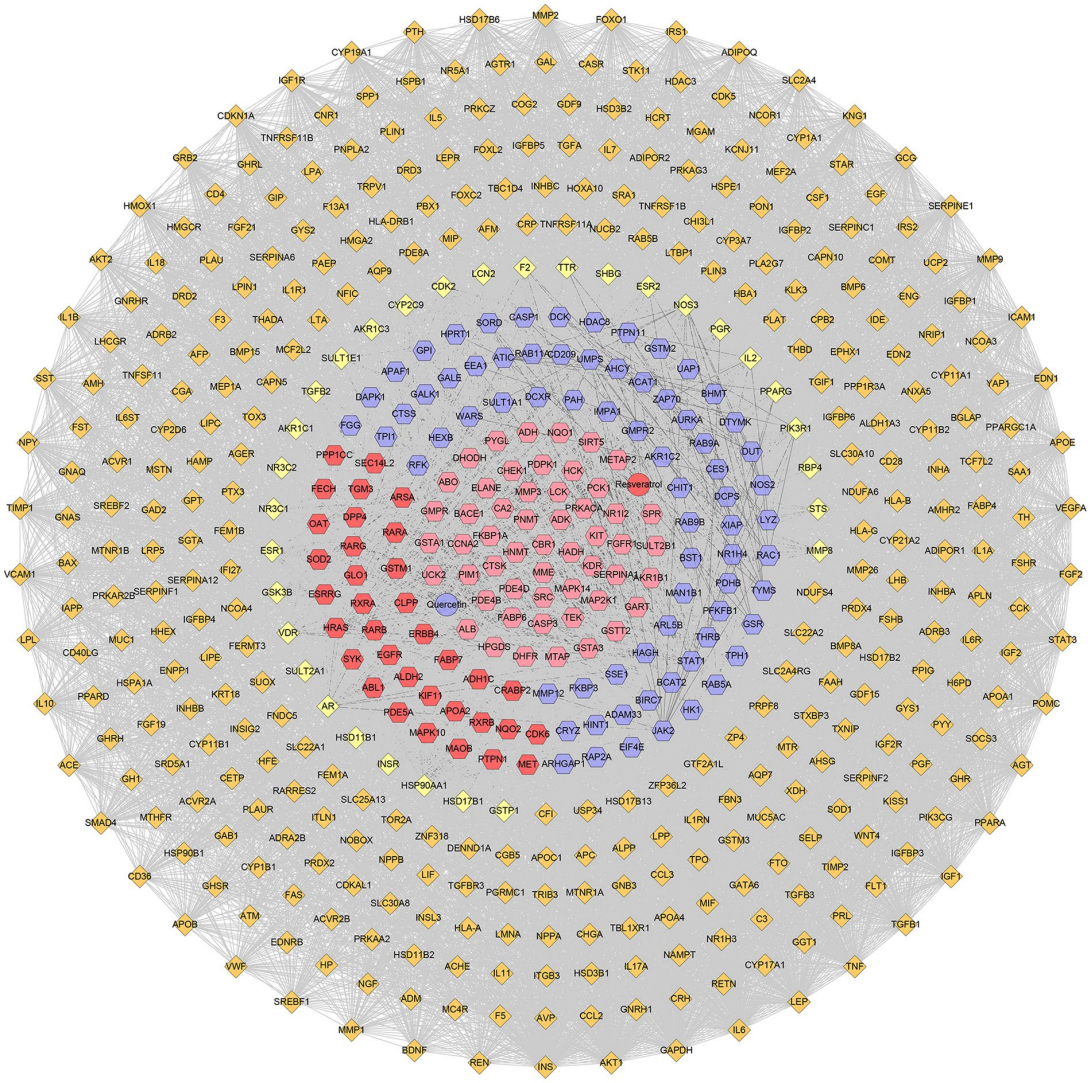
QRC-PCOS network (Blue and red hexagons stand for compound targets of resveratrol and quercetin, resp. Pink hexagons stand for common compound targets of both resveratrol and quercetin. Red and blue circles stand for resveratrol and quercetin, resp. Yellow diamonds stand for QRS-PCOS targets. Orange diamonds stand for PCOS genes. Dark lines stand for the relation of compounds and other nodes, light lines stand for the relation of PCOS genes, QRS-PCOS target and compound targets).

Integrate PCOS genes and compound-potential target network, the QRC-PCOS PPI network was constructed. This network includes 548 nodes and 9017 edges (Fig. 9).

#### Cluster of QRC-PCOS PPI Network

**Figure 10:**
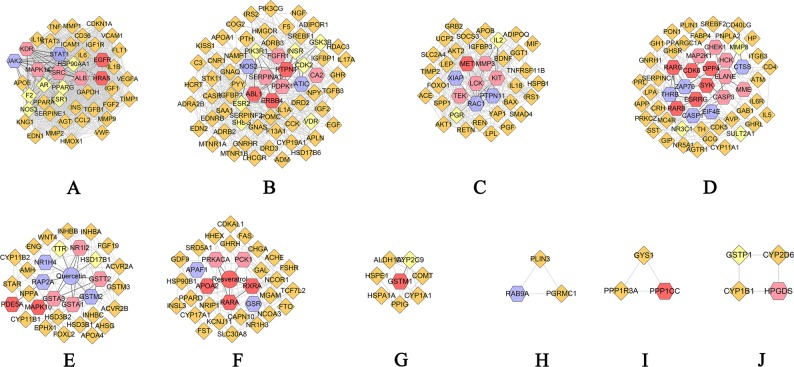
Cluster of QRC-PCOS PPI network (A, B, C, D… stand for cluster 1, 2, 3, 4…Blue and red hexagons stand for compound targets of resveratrol and quercetin, resp. Pink hexagons stand for common compound targets of both resveratrol and quercetin. Red and blue circles stand for resveratrol and quercetin, resp. Yellow diamonds stand for QRS-PCOS targets. Orange diamonds stand for PCOS genes. Dark lines stand for the relation of compounds and other nodes, light lines stand for the relation of PCOS genes, compound- PCOS target and compound targets).

The QRC-PCOS PPI network was analyzed by MCODE plugin in Cytoscape, and 10 clusters were obtained. In these clusters, cluster 5 gets quercetin, while cluster 6 has resveratrol. These two clusters may play an important role in the treatment of PCOS (Table 5 and Fig. 10).

**Table 5:** Cluster of QRC-PCOS PPI network.

Cluster	Score	Nodes	Edges	Genes
1	37.628	44	809	KDR, APOE, AR, MAPK14, STAT1, CCL2, HMOX1, FLT1, INS, TIMP1, IGF1, MMP9, CD36, JAK2, KNG1, TNF, STAT3, EDN1, ESR1, F2, CDKN1A, GAPDH, HSP90AA1, ALB, IL6, FGF2, SERPINE1, VCAM1, VWF, VEGFA, IL10, IL1B, MMP2, NOS3, IGF1R, HRAS, AGT, PPARA, ICAM1, TGFB1, PPARG, MMP1, SRC, EGFR
2	10.738	66	349	F13A1, LHCGR, FGFR1, CNR1, CDK2, NAMPT, TGFB3, PDPK1, IL17A, DRD2, COG2, INSR, SHBG, PTPN1, EDNRB, CASR, IRS2, HDAC3, ESR2, KISS1, CA2, ABL1, DRD3, ERBB4, GNRHR, HSD17B6, NPY, NOS2, PIK3R1, ADIPOR1, IL1A, VDR, SERPINF2, APLN, GSK3B, CYP19A1, PTH, IGFBP1, SREBF1, SAA1, GNAS, ADRB3, GHR, MTNR1A, ADRA2B, POMC, CCK, ATIC, NGF, IGF2, C3, HCRT, EDN2, GNAQ, ADM, SERPINA1, IGFBP2, PYY, PIK3CG, STK11, APOA1, EGF, F5, MTNR1B, ADRB2, HMGCR
3	10.432	38	193	GGT1, LPL, RAC1, BAX, AKT1, HSPB1, RETN, MIF, LEP, ADIPOQ, TIMP2, ACE, BDNF, YAP1, UCP2, IRS1, TNFRSF11B, SPP1, MMP3, MET, TEK, AKT2, REN, SMAD4, LCK, PGR, SLC2A4, PTPN11, APOB, KIT, FOXO1, IL18, IGFBP3, PGF, XIAP, GRB2, IL2, SOCS3
4	5.698	54	151	MAP2K1, SERPINC1, GCG, CD40LG, ELANE, NR5A1, AGTR1, MME, PNPLA2, TH, HCK, CD4, CDK5, CDK6, CASP1, SYK, ESRRG, IAPP, ATM, CASP3, PLIN1, PPARGC1A, FABP4, RARB, CRH, DPP4, RARG, SULT2A1, MMP8, MC4R, PRL, GHSR, IL5, IL6R, EIF4E, CYP11A1, GNRH1, HP, ITGB3, CTSS, CHEK1, GH1, SST, PON1, GHRL, SREBF2, PRKCZ, ZAP70, GIP, NR3C1, AVP, GAB1, THRB, LPA
5	4.903	32	76	Quercetin, GSTA1, EPHX1, HSD17B1, ACVR2B, INHBA, NPPA, NR1I2, AMH, INHBC, FGF19, RAP2A, HSD3B2, TTR, STAR, ENG, GSTT2, AHSG, FOXL2, PDE5A, MAPK10, CYP11B2, CYP11B1, GSTA3, INHBB, GSTM2, WNT4, GSTM3, APOA4, NR1H4, HSD3B1, ACVR2A
6	4.75	33	77	SRD5A1, RXRA, GHRH, RARA, GSR, MGAM, KCNJ11, NRIP1, ACHE, PCK1, APAF1, CDKAL1, NR1H3, FST, CYP17A1, GDF9, CHGA, FSHR, CAPN10, Resveratrol, FTO, FAS, GAL, TCF7L2, HSP90B1, HHEX, NCOA3, INSL3, SLC30A8, APOA2, PRKACA, NCOR1, PPARD
7	3	5	6	FSHB, INHA, ACVR1, AMHR2, CGA
8	3	3	3	RAB9A, PLIN3, PGRMC1
9	3	3	3	GYS1, PPP1CC, PPP1R3A
10	2.667	4	4	CYP2D6, CYP1B1, GSTP1, HPGDS

Meanwhile, several PCOS genes were also included in clusters. Cluster 1 includes CD36, MMP9, IGF1, TIMP1, INS, FLT1, HMOX1, CCL2, APOE, MMP1, TGFB1, ICAM1, PPARA, AGT, IGF1R, MMP2, IL1B, IL10, VEGFA, VWF, VCAM1, SERPINE1, FGF2, IL6, GAPDH, CDKN1A, EDN1, STAT3, TNF, KNG1, AR, PPARG, NOS3, HSP90AA1, F2, ESR1. Cluster 2 has TGFB3, NAMPT, CNR1, LHCGR, F13A1, HMGCR, ADRB2, MTNR1B, F5, EGF, STK11, APOA1, PIK3CG, PYY, IGFBP2, ADM, GNAQ, EDN2, HCRT, C3, IGF2, NGF, CCK, POMC, ADRA2B, MTNR1A, GHR, ADRB3, GNAS, SAA1, SREBF1, IGFBP1, PTH, CYP19A1, SERPINF2, APLN, IL1A, ADIPOR1, NPY, HSD17B6, GNRHR, DRD3, KISS1, HDAC3, CASR, EDNRB, IRS2, COG2, IL17A, DRD2, CDK2, GSK3B, VDR, PIK3R1, ESR2, SHBG, INSR. Cluster 3 includes TIMP2, ADIPOQ, LEP, MIF, RETN, HSPB1, BAX, AKT1, LPL, GGT1, SOCS3, GRB2, PGF, IGFBP3, IL18, FOXO1, APOB, SLC2A4, SMAD4, REN, AKT2, SPP1, TNFRSF11B, IRS1, UCP2, YAP1, BDNF, ACE, IL2, PGR. Cluster 4 gets CD40LG, GCG, SERPINC1, LPA, GAB1, AVP, GIP, PRKCZ, SREBF2, GHRL, PON1, SST, GH1, ITGB3, HP, GNRH1, CYP11A1, IL6R, IL5, GHSR, PRL, MC4R, CRH, FABP4, PPARGC1A, PLIN1, ATM, IAPP, CDK5, CD4, TH, PNPLA2, AGTR1, NR5A1, NR3C1, MMP8, SULT2A1. Cluster 5 has ACVR2A, HSD3B1, GSTM3, APOA4, WNT4, INHBB, CYP11B1, CYP11B2, AHSG, FOXL2, ENG, STAR, FGF19, HSD3B2, INHBC, AMH, NPPA, INHBA, ACVR2B, EPHX1, TTR, HSD17B1. Cluster 6 includes ACHE, NRIP1, KCNJ11, MGAM, GHRH, SRD5A1, PPARD, NCOR1, SLC30A8, INSL3, NCOA3, HHEX, HSP90B1, TCF7L2, GAL, FAS, FTO, CAPN10, CHGA, FSHR, GDF9, CYP17A1, FST, NR1H3, CDKAL1. Cluster 7 gets CYP1A1, COMT, HSPA1A, PPIG, HSPE1, ALDH1A3, CYP2C9. Cluster 8 includes PGRMC1, PLIN3. Cluster 9 gets GYS1, PPP1R3A. Cluster 10 includes GSTP1, CYP1B1, CYP2D6. The PCOS genes included by clusters may play a crucial role in the PCOS disease network, and may be the critical ones for the QRC treatment of PCOS.

The genes in clusters were analyzed by DAVID to obtain their biological processes. After the GO enrichment analysis, cluster 8 failed to return any biological processes. Cluster 9 does not return PCOS related biological processes. Since cluster 1 has the largest number of biological processes, and clusters 5 and 6 contain quercetin and Resveratrol, respectively, clusters 1, 5 and 6 are used as examples to demonstrate biological processes:

The GO IDs of biological processes in cluster 1 are GO:0008284, GO:0043066, GO:0051092, GO:0071392, GO:0071356, GO:0032755, GO:0006954, GO:0043627, GO:0071347, GO:0032869, GO:0032757, GO:0032868, GO:0043154, GO:0032355, GO:0070102, GO:0007179, GO:0043568, GO:0042346, GO:0043123, GO:0032715, GO:0032570, GO:0043124. Cluster 5 gets these biological processes: GO:0006694, GO:0046881, GO:0006703, GO:0006702, GO:0001541, GO:0060279, GO:0046882, GO:0061370, GO:0030509, GO:0071372, GO:0048599, GO:0071560, GO:0043401. And cluster 6 has: GO:0032024, GO:0043401, GO:0071392, GO:0043627, GO:0032869, GO:0042448, GO:0006702, GO:0008283, GO:0030073, GO:0006694, GO:0030521, GO:0071333.

The biological process corresponding to GO IDs can be found in Table S8. Information on the biological processes of other clusters, as well as genes contained in biological processes, can be found in Table S8.

In summary, ten clusters and fifty-nine biological processes were totally obtained based on QRC-PCOS network analysis. These biological processes may be closely related to the treatment of PCOS, such as influencing thalamus - pituitary - ovarian axis and hyperandrogenism (GO: 0071333, GO: 0006702, GO: 0006694, *et al*.), relieving hyperinsulinemia and insulin resistance (GO: 0071333, GO: 0032869, GO: 0032024, *et al*.), and so on.

#### Pathway of QRC-PCOS PPI Network

**Figure 11:**
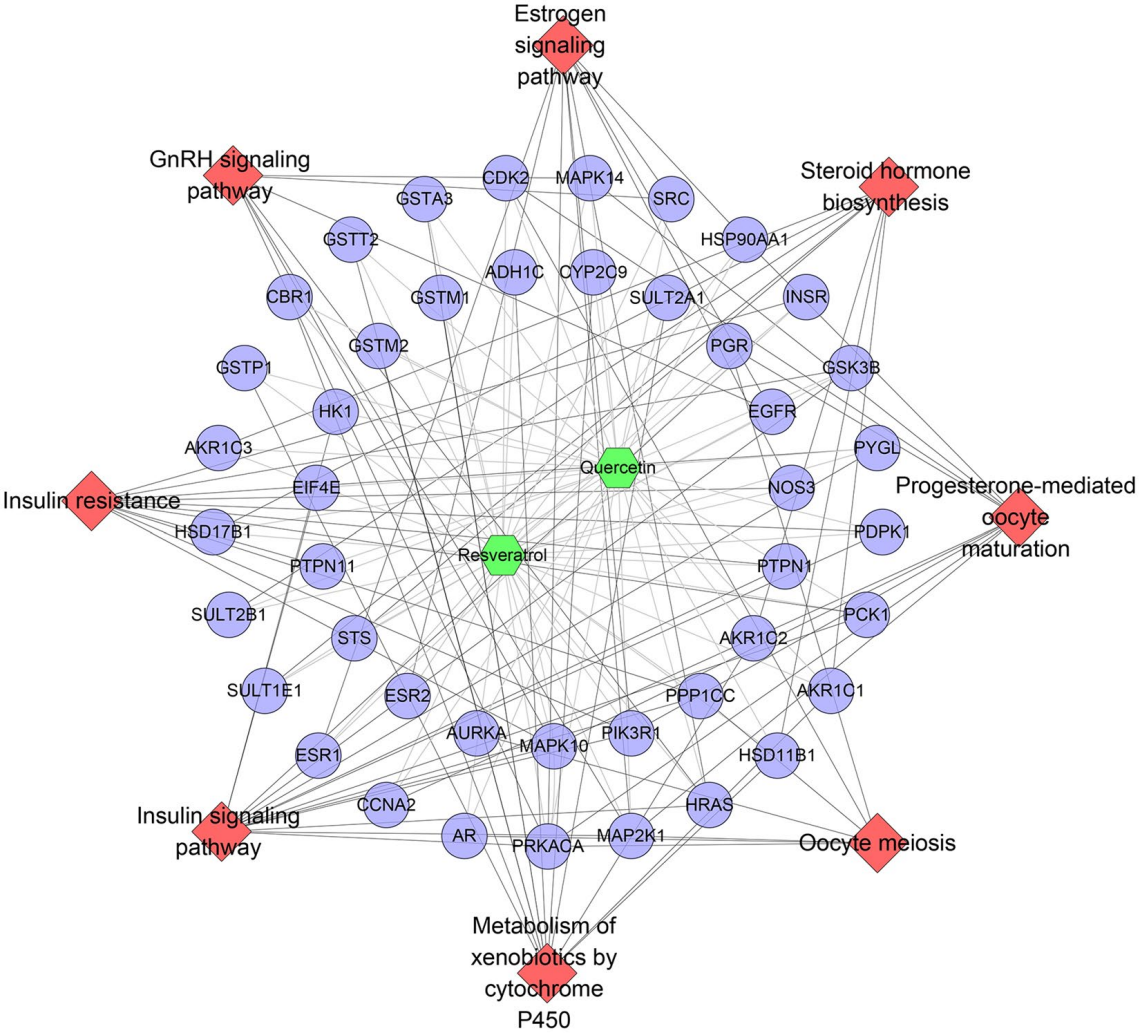
Pathway of QRC-PCOS PPI network (Blue circles stand for compound targets. Red diamonds stand for pathways. Green hexagons stand for compounds).

All targets were put into DAVID to undergo pathway enrichment analysis. After that, eight PCOS-related signaling pathways were acquired (Fig. 11). In Fig. 11, insulin signaling pathway has the most targets (14 proteins); metabolism of xenobiotics by cytochrome P450 has 12 targets; insulin resistance has 11 targets; estrogen signaling pathway has 10 targets; progesterone-mediated oocyte maturation has 9 targets and so on. These signaling pathways may play a crucial role in the treatment of PCOS with QRC. The details of each signaling pathway were shown in Table S7.

#### QRC-PCOS-Other human proteins PPI network analysis

**Figure 12:**
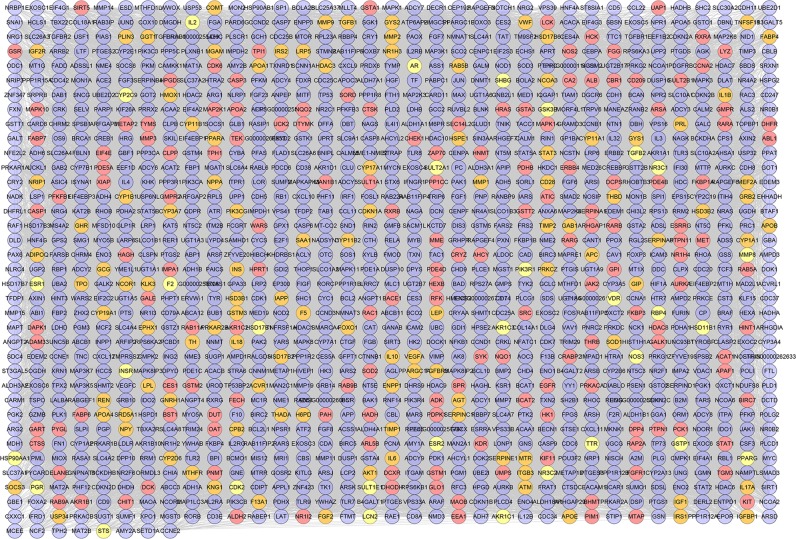
QRC-PCOS-other human proteins PPI network (Blue, pink, orange and yellow circles stand for other human protein compound targets, PCOS genes and QRS-PCOS targets, resp).

Due to that the network is built with potential targets only, it has some limitations. Therefore, we collect the QRC-associated other human proteins from String to construct this network (Fig. 12). It consists of 1268 nodes and 35438 edges, and is a expand network of QRC-PCOS PPI network. Among other human proteins, some PCOS genes and QRC-PCOS targets (orange and yellow nodes) were found, suggesting that QRC can directly or indirectly regulate the PCOS gene to achieve therapeutic effects. The targets are shown in Table S9.

**Figure 13:**
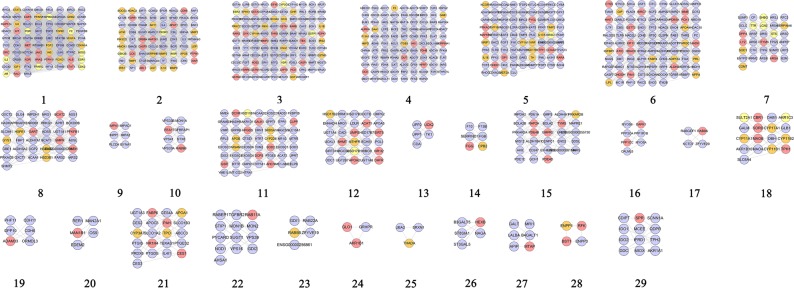
Cluster of QRC-PCOS-other human proteins PPI network (Blue, pink, orange and yellow circles stand for other human protein compound targets, PCOS genes and QRS-PCOS targets, resp).

The QRC-PCOS-other human proteins PPI network was analyzed by MCODE plugin in Cytoscape, and 28 clusters were obtained (Fig. 13). The genes in clusters were analyzed by DAVID to obtain their biological processes. After the GO enrichment analysis, cluster 17 did not return biological processes; and cluster 9, 10, 13, 14, 16, 19, 20, 23, 24, 25, 26, 27, 28, 29 failed to return PCOS-related biological processes. Cluster 9 did not return PCOS related biological processes. Since cluster 1 has the largest number of biological processes, it is used as examples to demonstrate biological processes:

The GO IDs of biological processes in cluster 1 are GO:0008284, GO:0043066, GO:0032355, GO:0043627, GO:0032869, GO:0001541, GO:0032868, GO:0051092, GO:0032570, GO:0043401, GO:0006978, GO:0050729, GO:0008286, GO:0006954, GO:0071392, GO:0001547, GO:0030521, GO:1990314, GO:0001542, GO:0050727, GO:0043123, GO:0032731, GO:0043568, GO:0048599, GO:0071391.

The biological process corresponding to GO IDs can be found in Table S11. Information on the biological processes of other clusters, as well as genes contained in biological processes, can be found in Table S11.

This network is an extension of the previous; it is built to observe the relationship between potential targets, PCOS genes, and other human proteins. Based on this network, we not only found the same biological processes as previous, but also discovered new biological processes related to PCOS, such as GO: 0001547, GO: 0001542, GO: 2001275, GO: 0002690, *et al*. This is a supplement to the QRC-PCOS PPI network, which allows us to better understand the mechanism by which QRC treats PCOS.

**Figure 14:**
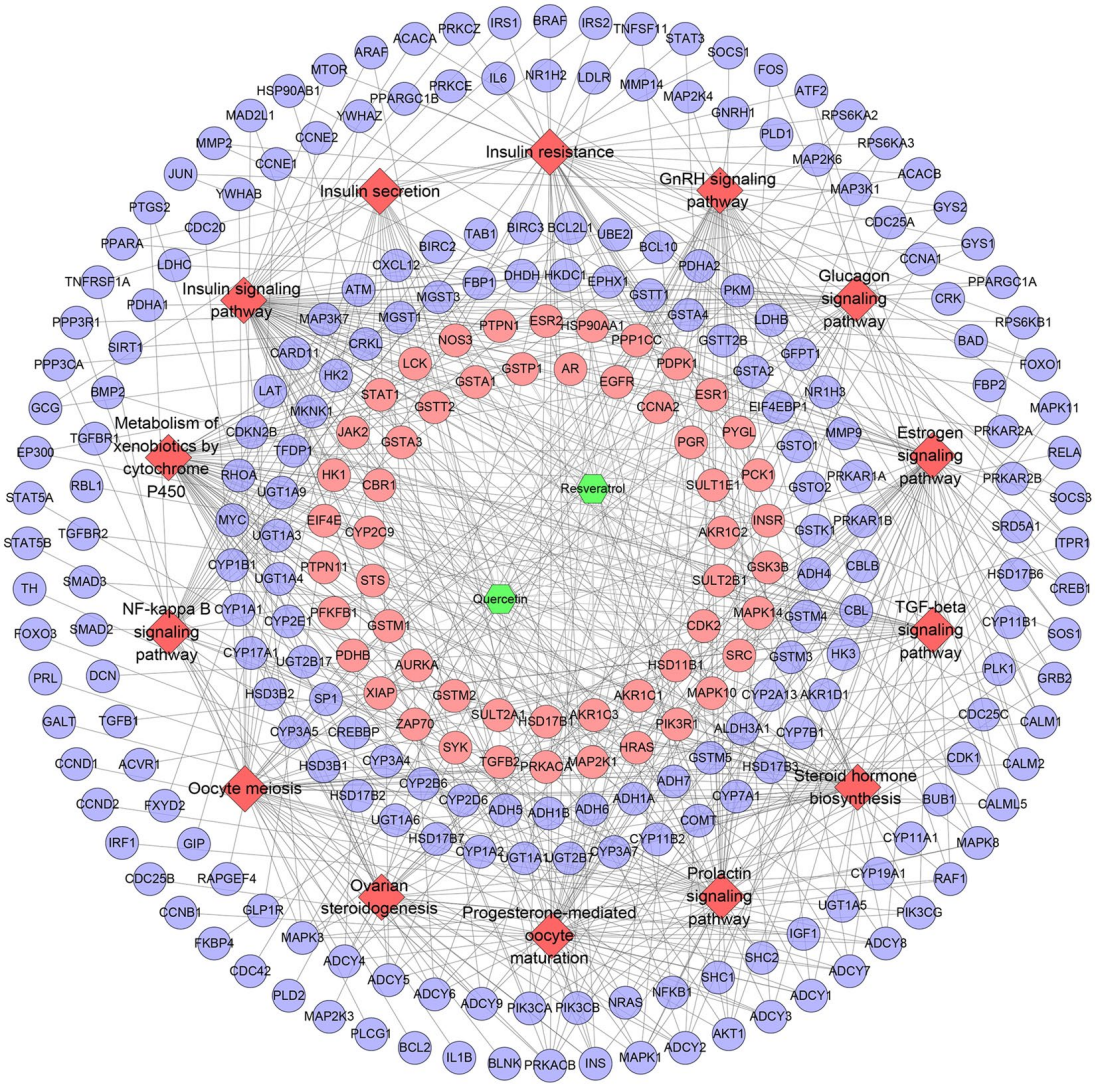
Pathway of QRC-PCOS-other human proteins PPI network (Blue and pink circles stand for other human proteins and compound targets, resp. Red diamonds stand for pathways. Green hexagons stand for compounds).

Meanwhile, we get fourteen PCOS related pathways. Fifty-three potential targets and two hundred and sixteen other human proteins are included in this network. Insulin signaling pathway contains 63 genes, which is the most; metabolism of xenobiotics by cytochrome P450 gets 50 genes; estrogen signaling pathway includes 45 genes; progesterone-mediated oocyte maturation has 44 genes; prolactin signaling pathway get 43 genes; insulin resistance contains 42 genes; GnRH signaling pathway has 40 genes and so on (Fig. 14). The details are described in Table S12.

In this network, these pathways contain some one that has appeared in Fig. 11, which indicate that this network is a confirmation, complement and expansion for the previous one. This suggests that QRC may directly or indirectly regulate PCOS genes and its related potential targets to affect the PCOS-associated pathways so as to achieve treatment effect.

**Figure 15:**
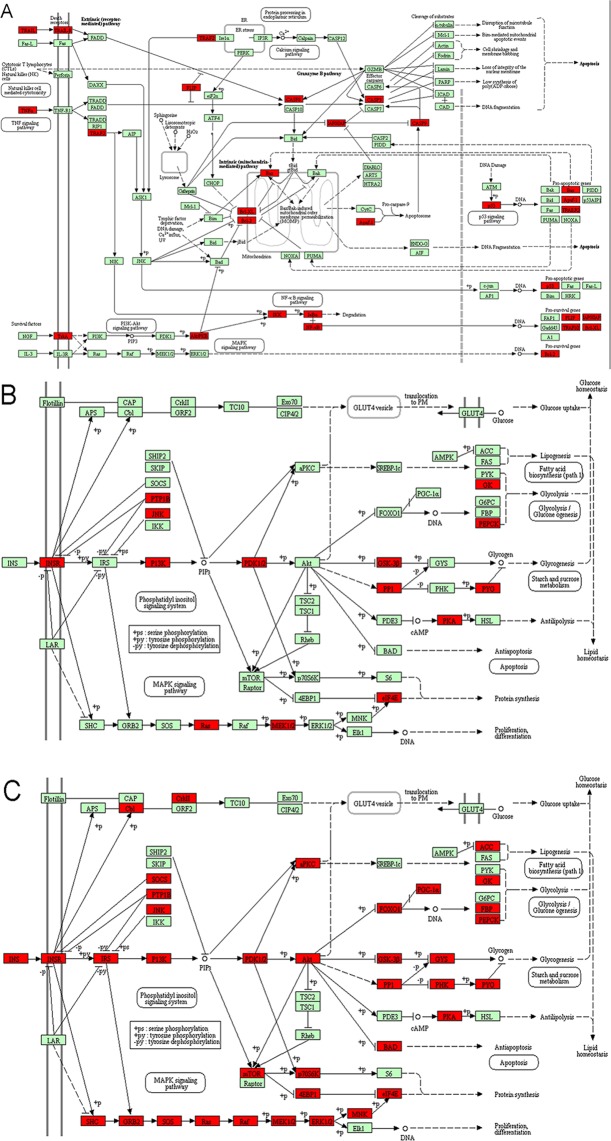
(**A**) Apoptosis adapted from KEGG (ID: hsa04210), the know targets were signed in red; (**B**,**C**) the insulin signaling pathway adapted from KEGG (ID: hsa04910) the predicted targets and other human proteins were signed in red.

For QRC’s network, the apoptosis in Fig. 4, and the insulin signaling pathway in 11 and 14 contain the highest number of targets; these targets and genes are shown in Fig. 15 (red squares) as an example. It can be found that some of the red squares in Fig. 15B overlap with those in Fig. 15C. QRC may regulate signaling pathways by regulating these targets. Figure 15 is adapted from KEGG (ID: hsa04210 and hsa04910)^48,49^.

As a reproductive and metabolic hormone, insulin acts as a co-gonadotropin through its cognate receptors, regulating ovarian steroid production. Human studies have confirmed that hyperinsulinemia increases androgen production in PCOS. Insulin is also a major regulator of SHBG production. Reducing insulin resistance can also recover ovulatory cycles. These findings result in insulin-sensitizing drugs become the important therapeutic modality for PCOS^50,51^.

In the central nervous system, insulin signaling is also vital for ovulation. These findings will be directly translated into a new therapy for PCOS with insulin- sensitizing drugs. In addition, androgens also have important effects on insulin sensitivity and secretion. A number of recent studies have shown that resveratrol and quercetin can improve insulin resistance, hyperandrogenism and so on^11,12,14,15,52^. This research also shows that in insulin resistance and hyperandrogenism, QRC improves hyperinsulinemia, hyperandrogenemia and steroid hormone abnormalities, which eventually improves the metabolism of PCOS. After an in-depth study of QRC’s related biological network, more insulin and sex hormone-related signaling pathways and biological processes were discovered, such as: (GO:0032869) cellular response to insulin stimulus, (GO:0033686) positive regulation of luteinizing hormone secretion, (GO:0048009) insulin-like growth factor receptor signaling pathway, (GO:0061370) testosterone biosynthetic process, (hsa04917) prolactin signaling pathway. This indicates the therapeutic effect of QRC on PCOS.

In terms of inflammation, latest research shows that most patients with PCOS, approximately 50–70%, experience the varying degrees of insulin resistance and mild diffuse inflammation^7^. The chronic inflammation (besides hyperandrogenism and insulin resistance), especially mild chronic inflammation, is a hallmark of PCOS in the microenvironment of patients^3^. The gene polymorphism of IL-1α, not IL-1β, relates to PCOS^53^. Meanwhile, current research shows quercetin and resveratrol can improve the ovarian inflammation and oxidative stress of PCOS^10,13^. In addition, resveratrol can inhibit the expression of TNF-α and IL-6 mRNA in the visceral subcutaneous adipose tissue of PCOS rats, thereby inhibiting the inflammatory response^54^. The results of in-depth analysis also revealed some new anti-inflammatory related signaling pathways and biological processes, such as: (GO:0071347) cellular response to interleukin-1, (GO:0006954) inflammatory response, (GO:0032753) positive regulation of interleukin-4 production, (GO:0007179) transforming growth factor beta receptor signaling pathway, (hsa04064) NF-kappa B signaling pathway, (hsa04350) TGF-beta signaling pathway.

Ovarian interstitial cell apoptosis has been confirmed to be closely related to the severity and development of PCOS^3^. Interstitial cells play an important role in maintaining androgen synthesis and metabolic balance, which is the basis of changes in ovarian structure^28^. Therefore, causing interstitial cell apoptosis or inhibition may be a potential strategy for the treatment of PCOS. Current study has shown that resveratrol promotes apoptosis to reduce rat theca-interstitial cell growth *in vitro*, inhibits insulin-induced rat theca-interstitial cell and reduces the expression of vascular endothelial growth factor (VEGF)^55^. The results of in-depth analysis also show the effects of QRC on ovary-related signaling pathways and biological processes, such as: (GO:0001541) ovarian follicle development, (GO:0048599) oocyte development, (hsa04114) oocyte meiosis.

Current experimental studies also partially verified the effects of quercetin or resveratrol. In terms of insulin resistance, current studies have shown that resveratrol inhibits lipid peroxidation and insulin resistance by interfering with serum MDA (p = 0.034) and HOMA-IR^55^; while quercetin significantly reduced insulin resistance and inhibited the expression of uterine GLUT4 and ERα gene in PCOS rats, thereby improving PCOS and its complications^56^. In terms of anti-oxidative stress, resveratrol in combination with metformin can induce weight gain, hormone distribution and ovarian follicular cell structure by inducing antioxidant and anti-inflammatory systems through SIRT1 and AMPK activation in PCOS^57^. In terms of angiogenesis, resveratrol may improve certain outcomes in patients with PCOS by altering the serum levels of certain sex hormones and the expression of VEGF and HIF1 genes in the granule cell angiogenesis pathway^58^. In regulating hormones, quercetin can reduce resistin levels, as well as testosterone and LH concentrations in patients with PCOS^59^. In animal models, quercetin relieves hormone and metabolic disorders in PCOS animals by lowering testosterone, estradiol, and progesterone levels^60^. Additionally, several studies have achieved certain effects through the intervention of QRC in the obese rat model caused by the high-fat diet^61–66^. Since obesity can cause metabolic syndrome and is associated with PCOS, we think that QRC can also be used for the treatment of PCOS. Zhao *et al*. found that combination of resveratrol (15 mg/kg per day) and quercetin (30 mg/kg per day) can reduce serum TNF-α, IL-6 and MCP-1, as well as obesity-induced inflammation syndrome^61^. This study also examined metabolic parameters after 10 weeks^61^. Another study showed that such dose of QRC can effectively reduce serum insulin levels and insulin resistance^62^. Zhao *et al*. also found that administration of resveratrol (120 mg/kg per day) and quercetin (240 mg/kg per day) attenuated the expression of systemic pro-inflammatory adipokines, including leptin, TNF-α, monocyte chemotactic protein -1 and IL-6^63^. Arias *et al*. also showed the synergy between resveratrol (15mg/kg per day) and quercetin (30mg/kg per day). The intervention of QRC can affect the metabolic pathways involved in the accumulation of triacylglycerol in adipose tissue^64^. Meanwhile, in metabolic syndrome (MetS) rat model, the combination of quercetin (50 mg/kg per day) and resveratrol (0.95 mg/kg per day) significantly reduced insulin concentration and HOMA-IR in MetS rats^65^. It also increased adiponectin and leptin levels, and up-regulated SIRT1 and SIRT2 expression in white adipose tissue of MetS rats^65^. Another study showed that QRC treatment can modulate PPAR-mediated uncoupling protein (UCP-) 1, 2 and 3 expression in visceral white adipose tissue of the MetS rat model^66^.

Although this research did not perform experiments, it used a computer model to simulate QRC intervention in disease-related targets in patients with PCOS, which has certain advantages over animal experiments and cell experiments. The limitation of this study is that it did not conduct animal experiments, cell experiments and so on to verify the findings. However, these findings have been partially confirmed by published research. Meanwhile, the database used in computer simulations applied the pharmacophore model for molecular docking, and its reliability has been confirmed by several studies. Overall, although the QRC’s potential targets and their related other human proteins have been shown to have a therapeutic effect on PCOS through this network analysis, it still needs further research to make a further confirmation. Through this network relationship, we can conduct a thorough and in-depth study of the pharmacological and molecular mechanism of QRC. Through our research, we can find that insulin signaling pathway, metabolism of xenobiotics by cytochrome P450, GnRH signaling pathway, estrogen signaling pathway, progesterone-mediated oocyte maturation, prolactin signaling pathway, insulin resistance and so on may be the focus pathways of the future study of QRC (Figs. 4, 7, 11, 14).

## Conclusions

PCOS is a chaotic disease with multiple factors, such as thalamus - pituitary - ovarian axis abnormality, insulin resistance, *et al*., and various clinical manifestations, so we probably need a multi-drug combination with multi-target to against it. Since that quercetin and resveratrol have some good effects on PCOS, QRC may combine their efficacy and become a new multi-drug combination with multi-target. In this research, we applied a systematic pharmacological method that integrated multiple drug target prediction, and network analysis to predict the potential targets and get the reported targets and other human proteins. We not only compared the PCOS associated biological processes and pathways of resveratrol, quercetin and QRC, but also in depth explored the pharmacological and molecular mechanism of QRC. Hence, we have a deeper understanding and new insights into the chemical basis and pharmacological effects of these three, especially QRC. This study not only offers clues to the researcher who is interested in comparing the differences among resveratrol, quercetin and QRC, but also provides hints for the researcher who wants to explore QRC’s various synergies and its pharmacological and molecular mechanism.

## Supplementary information

Table S1

Table S2

Table S3

Table S4

Table S5

Table S6

Table S7

Table S8

Table S9

Table S10

Table S11

Table S12

## Data Availability

The datasets generated during and/or analyzed during the current study are available in the TCMSP repository, http://ibts.hkbu.edu.hk/LSP/tcmsp.php; PharmMapper repository, http://lilab-ecust.cn/pharmmapper; Genecards repository, http://www.genecards.org; OMIM repository, http://omim.org/; String repository, http://string-db.org; DAVID repository, https://david-d.ncifcrf.gov.
