# Publisher Correction: Exploring the Pharmacological Mechanism of Quercetin-Resveratrol Combination for Polycystic Ovary Syndrome: A Systematic Pharmacological Strategy-Based Research

**DOI:** 10.1038/s41598-020-59780-z

**Published:** 2020-02-14

**Authors:** Kailin Yang, Liuting Zeng, Tingting Bao, Zhiyong Long, Bing Jin

**Affiliations:** 1 Department of Cardiac Surgery, Beijing Anzhen Hospital, Capital Medical University, Beijing, China; 2 Capital Medical University, Beijing, China; 3 Xiyuan Hospital, China Academy of Chinese Medical Sciences, Beijing, China; 4 School of Clinical Medicine (Xiyuan Hospital), Beijing University of Chinese Medicine, Beijing, China; 5 Shantou University Medical College, Shantou University, Shantou, Guangdong China

Correction to: *Scientific Reports* 10.1038/s41598-019-54408-3, published online 05 December 2019

The original PDF version of this Article contained an error in the order of corresponding authors. This has now been corrected in the PDF version of this Article.

Additionally, the Author Contributions section in this Article was incorrect.

“Liuting Zeng and Kailin Yang dominate the concept and carry out a comprehensive design; Tingting Bao and Bing Jin participant in the concept and design. Kailin Yang, Liuting Zeng, Tingting Bao and Zhiyong Long are responsible for data analysis and interpretation. Liuting Zeng, Kailin Yang, Tingting Bao drafted the paper; Bing Jin supervised the study; all authors participated in the analysis and interpretation of data and approved the final paper. Kailin Yang, Liuting Zeng and Tingting Bao should be considered joint first author.”

now reads:

“Liuting Zeng and Kailin Yang dominate the concept and carry out a comprehensive design; Tingting Bao and Bing Jin participant in the concept and design. Kailin Yang, Liuting Zeng, Tingting Bao and Zhiyong Long are responsible for data analysis and interpretation. Liuting Zeng, Kailin Yang, Tingting Bao drafted the paper; Bing Jin supervised the study; all authors participated in the analysis and interpretation of data and approved the final paper. Kailin Yang, Liuting Zeng and Tingting Bao should be considered joint first author. Bing Jin is the first corresponding author because he supervised the study.”

